# Supplementary figures and images for: Maturing neutrophils of lower density associate with thrombocytopenia in Puumala orthohantavirus-caused hemorrhagic fever with renal syndrome
Source: Front Immunol. 2024 Jul 1;15:1419787. doi: 10.3389/fimmu.2024.1419787 (PMC11246883; doi:10.3389/fimmu.2024.1419787)

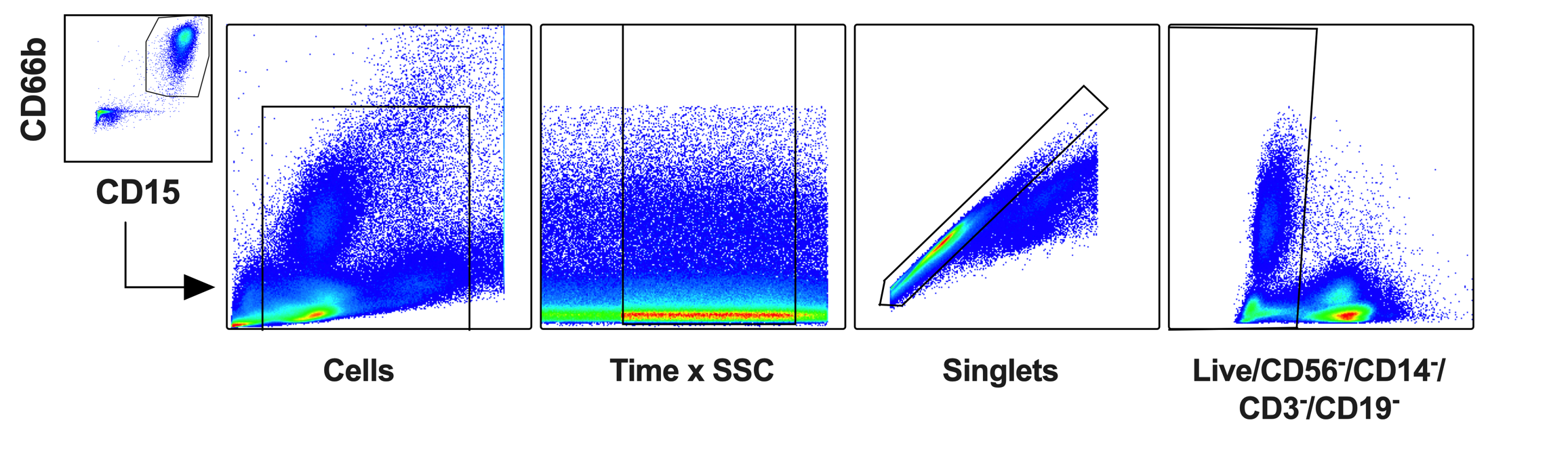

Supplement: Supplementary Figure 1 — Flow cytometry gating strategy. Single cells were gated after the exclusion of debris and a time gate, which was employed for the selection of a steady collection flow to ensure proper sample quality and homogeneity. Then, cells negative to CD3, CD56, CD14, CD19 and the dead cell marker were gated in, from which CD66b/CD15 double positive cells were finally labeled as LDGs. [file Image_1.tiff]

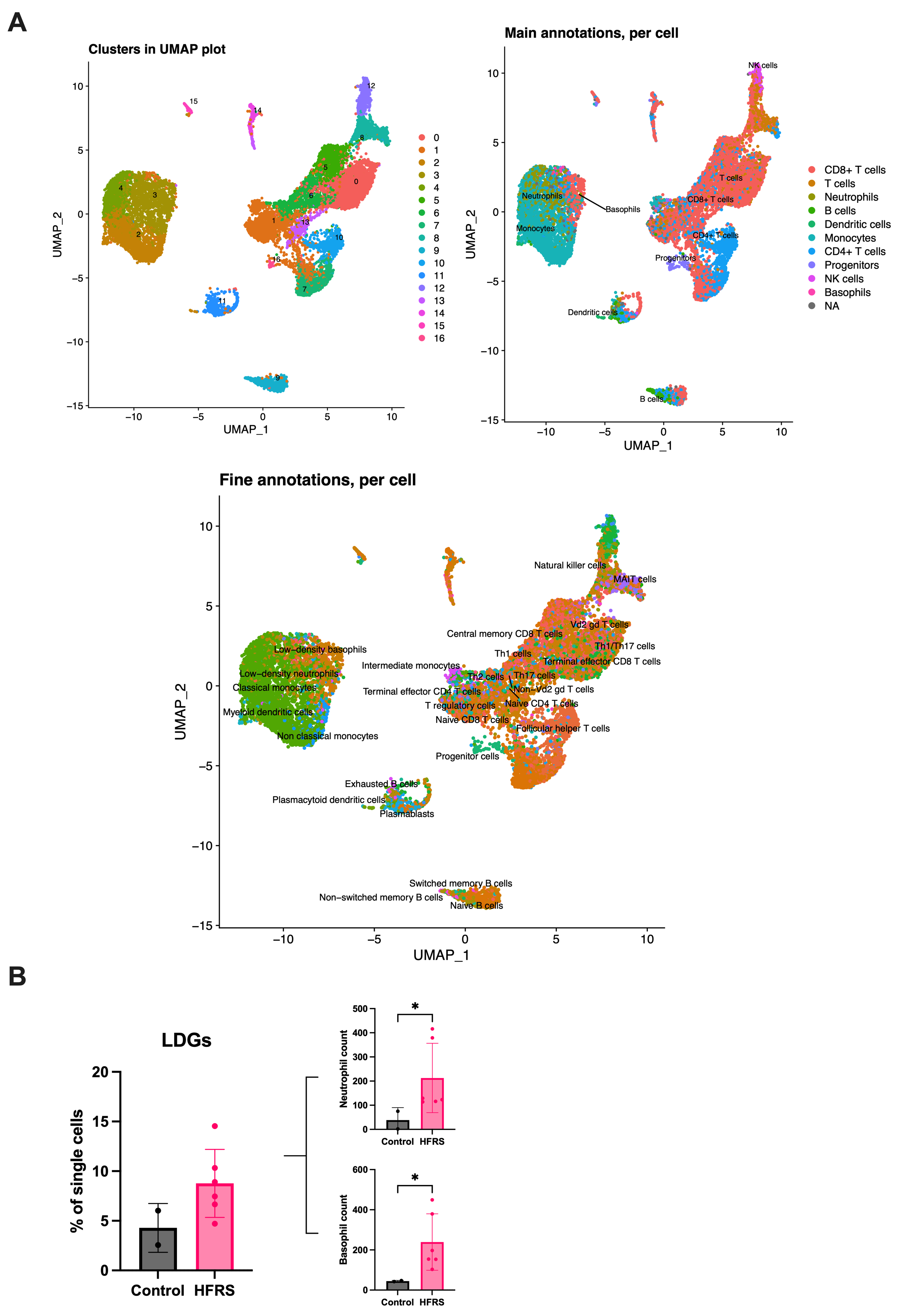

Supplement: Supplementary Figure 2 — Increase of LDGs in Hantaan orthohantavirus-caused HFRS identified by single cell RNA sequencing analysis. (A) UMAP visualization panels depicting the identified numbered clusters. The main annotations are labeled in the UMAP plot using SingleR in Chipster, and the fine annotations are presented, providing additional insights with more clusters and labels. (B) Bar plot representing the percentage of LDGs among the total single cells identified by single cell RNA sequencing analysis (scRNA-seq) in both control and HFRS groups. Two smaller bar plots depict the counts of single cells classified as low-density neutrophils and low-density basophils. [file Image_2.tiff]

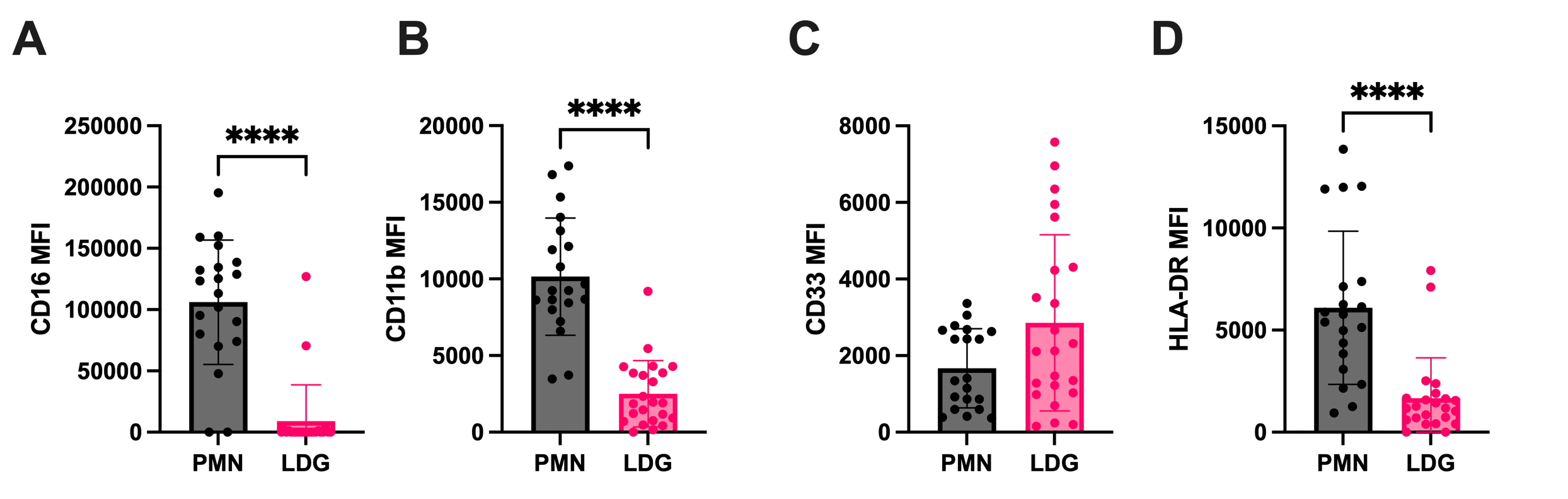

Supplement: Supplementary Figure 3 — Median fluorescent intensity of CD16, CD11b, CD33 and HLA-DR in PUUV LDGs and PMNs. The median fluorescence intensity (MFI) of CD16 (A), CD11b (B), CD33 (C) and HLA-DR (D) as measured by flow cytometry were compared between PUUV LDGs and PMNs. *p < 0.05, **p < 0.01, ***p < 0.001. P values were calculated with Mann-Whitney test. [file Image_3.tiff]

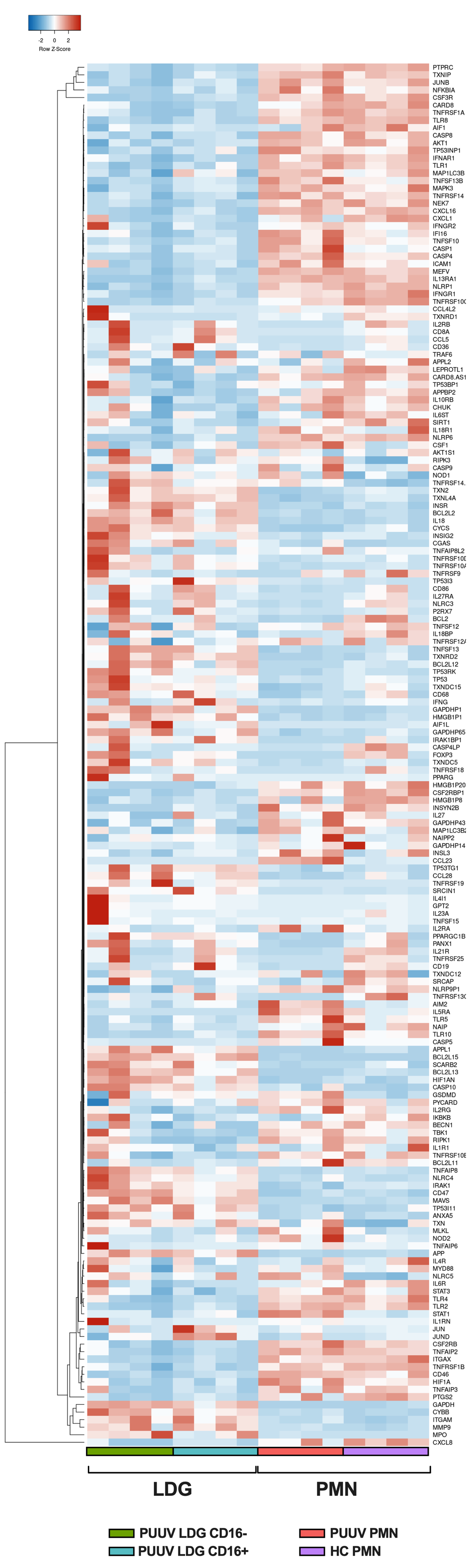

Supplement: Supplementary Figure 4 — Gene expression of inflammasome-related genes in neutrophils during acute PUUV-HFRS. Heatmap of differentially expressed inflammasome-related genes between LDGs and PMNs. [file Image_4.tiff]

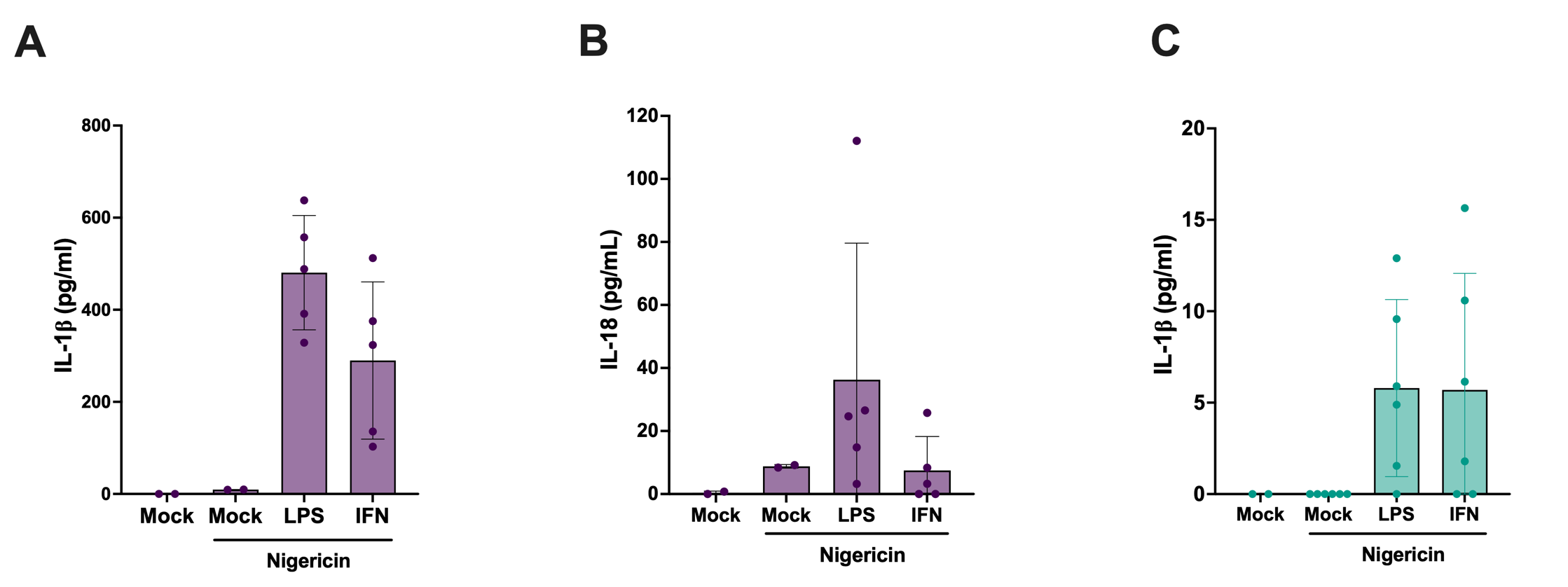

Supplement: Supplementary Figure 5 — Inflammasome activation in PUUV-HFRS neutrophils. (A) IL-1β and (B) IL-18 levels in PUUV LDGs, and (C) IL-1β levels in PUUV PMNs following interferon or LPS priming and nigericin activation. *p < 0.05. P values calculated with Kruskall-Wallis test. [file Image_5.tiff]
